# Deep Learning–Based COVID-19 Pneumonia Classification Using Chest CT Images: Model Generalizability

**DOI:** 10.3389/frai.2021.694875

**Published:** 2021-06-29

**Authors:** Dan Nguyen, Fernando Kay, Jun Tan, Yulong Yan, Yee Seng Ng, Puneeth Iyengar, Ron Peshock, Steve Jiang

**Affiliations:** ^1^ Medical Artificial Intelligence and Automation (MAIA) Laboratory, University of Texas Southwestern Medical Center, Dallas, TX, United States; ^2^ Department of Radiation Oncology, University of Texas Southwestern Medical Center, Dallas, TX, United States; ^3^ Department of Radiology, University of Texas Southwestern Medical Center, Dallas, TX, United States

**Keywords:** deep learning, generalizability, convolutional neural network, classification, computed tomography, COVID-19, SARS-CoV-2

## Abstract

Since the outbreak of the COVID-19 pandemic, worldwide research efforts have focused on using artificial intelligence (AI) technologies on various medical data of COVID-19–positive patients in order to identify or classify various aspects of the disease, with promising reported results. However, concerns have been raised over their generalizability, given the heterogeneous factors in training datasets. This study aims to examine the severity of this problem by evaluating deep learning (DL) classification models trained to identify COVID-19–positive patients on 3D computed tomography (CT) datasets from different countries. We collected one dataset at UT Southwestern (UTSW) and three external datasets from different countries: CC-CCII Dataset (China), COVID-CTset (Iran), and MosMedData (Russia). We divided the data into two classes: COVID-19–positive and COVID-19–negative patients. We trained nine identical DL-based classification models by using combinations of datasets with a 72% train, 8% validation, and 20% test data split. The models trained on a single dataset achieved accuracy/area under the receiver operating characteristic curve (AUC) values of 0.87/0.826 (UTSW), 0.97/0.988 (CC-CCCI), and 0.86/0.873 (COVID-CTset) when evaluated on their own dataset. The models trained on multiple datasets and evaluated on a test set from one of the datasets used for training performed better. However, the performance dropped close to an AUC of 0.5 (random guess) for all models when evaluated on a different dataset outside of its training datasets. Including MosMedData, which only contained positive labels, into the training datasets did not necessarily help the performance of other datasets. Multiple factors likely contributed to these results, such as patient demographics and differences in image acquisition or reconstruction, causing a data shift among different study cohorts.

## Introduction

Since the outbreak of the 2019 coronavirus disease (COVID-19) in December 2019, the total worldwide death count due to COVID-19 has exceeded a million (Pérez-Peña, 2020). COVID-19 can affect multiple organ systems and cause fever, flu-like symptoms, cardiovascular damage, and pulmonary injury. The most common manifestivation of COVID-19 upon initial presentation is pneumonia. While some patients are asymptomatic or have mild symptoms, a small percentage of patients may develop severe acute respiratory distress syndrome (ARDS) that requires intubation in the intensive care unit and is associated with poor prognosis. The mortality rate is over 60% once they progress to the severe illness stage (Guan et al., 2020). Since chest CTs are performed for reasons other than pulmonary symptoms as well, an automated tool that can opportunistically screen chest CTs for the disease can potentially be used to identify patients with COVID-19. First, it has been suggested that patients with COVID-19 when identified in the early stage can be treated to prevent progression to the later stage of the disease (McCullough, et al., 2020a; McCullough, et al., 2020b; FLARE, 2020). Second, identification of asymptomatic patients in the early stage using CT (Ali and Ghonimy, 2020) provides a time window during which they can isolate themselves to prevent the spread to others.

Several efforts around the world have been focused on the identification or categorization of COVID-19–positive patients according to their various types of medical data. As part of the effort to understand and control this disease, large COVID-19 datasets of different formats have been curated and publicly released around the world. One group of studies focuses on using artificial intelligence (AI) technologies, in particular deep learning (DL)–based models, to detect COVID-19 through chest radiography and computed tomography (CT). These studies found high accuracy rates ranging from 82 to 98% (Wang L. et al., 2020; Sethy et al., 2020; Narin et al., 2021; Apostolopoulos and Mpesiana, 2020; Hemdan et al., 2020; Wang S. et al., 2020; Xu et al., 2020; Ozturk et al., 2020; Shibly et al., 2020; Oh et al., 2020; Jin et al., 2020). The high accuracy rates are promising and encourage the use of this technology in the clinical setting.

However, the generalizability of these models to other clinical settings around the world is not clear. The data usually found in clinical practice are often incomplete and noisy, and they may have high intra- and inter-study variability among different environments. This scenario often makes it difficult from a research perspective to develop algorithms and implement them in the clinic. Due to various restrictions on sharing patient data, many algorithms are developed with limited data that are specific to a clinic or a region. However, differences in several demographic factors, such as population distribution of race, ethnicity, and geography, can greatly impact the overall accuracy and performance of an algorithm in a different clinical setting (Topol, 2020). In addition, different methods of data collection by hospitals around the world may also impact an algorithm’s performance. Because the boom of DL technologies has happened only within the last several years, the number of studies testing the robustness and performance of AI algorithms across various clinical settings is extremely limited (Topol, 2020). Therefore, there is very little knowledge about how well a model will perform in a realistic clinical environment over time.

For example, Barish et al. (2021) demonstrated a particular public model developed by Yan (2020) that predicted mortality from COVID-19–positive patients—which performed well on an internal dataset with an accuracy of 0.878—failed to accurately predict the mortality on an external dataset, with an accuracy of only around 0.5. Another similar negative study applied Yan et al.’s model on an external dataset and drew similar conclusions about the accuracy of its mortality prediction (Quanjel et al., 2021). A systematic review of 107 studies with 145 prediction models was conducted, and the studies reported that all models had a high bias, due to nonrepresentative control datasets and overly optimistic reported performance (Wynants et al., 2020), which can additionally lead to unrealistic expectations among clinicians, policy makers, and patients (Laghi, 2020). Bachtiger et al. concluded that this boom of DL models for COVID-19 focused far too much on developing novel prediction models without a comprehensive understanding of its practical application and biases from the dataset (Bachtiger et al., 2020). Others have similarly concluded that AI has yet to have any impact on the prevailing pandemic and that extensive and comprehensive gathering of diagnostic COVID-19–related data will be essential to develop useful AI models (Naudé, 2020).

As part of the efforts to collect data, large datasets of 3D computed tomography (CT) scans with COVID-19–related labels have been publicly released. This provides an opportunity to study the generalizability of DL algorithms developed using these volumetric datasets. In this study, we collected and studied one internal dataset collected at UT Southwestern (UTSW) and three large external datasets from around the world: 1) China Consortium of Chest CT Image Investigation (CC-CCII) Dataset (China) (Zhang et al., 2020), 2) COVID-CTset (Iran) (Rahimzadeh et al., 2021), and 3) MosMedData (Russia) (Morozov et al., 2020). We trained DL-based classification models on various combinations of datasets and evaluated the model performance on the held-out test data from each of the datasets.

## Methods

### Data

We collected one internal dataset at UTSW and three large datasets from around the world that are publicly available—1) China Consortium of Chest CT Image Investigation (CC-CCII) Dataset (China), 2) COVID-CTset (Iran), and 3) MosMedDat (Russia)—which is summarized in Table 1. The UTSW dataset is composed of three subsets of anonymized imaging data obtained retrospectively. The study protocol was approved by the institutional review board and the requirement for informed consent was waived. The first subset includes patients who tested positive for severe acute respiratory syndrome coronavirus 2 on real-time polymerase chain reaction between March and November 2020 and who had a chest CT scan performed within the first 7 days of diagnosis. All chest CT scans were obtained according to the standard clinical care—common clinical indications were to assess the worsening respiratory status and to rule out pulmonary thromboembolism. Chest CT is not obtained as a first-line modality to diagnose or screen for COVID-19 at UTSW. As such, the collected dataset had a mixture of contrast-enhanced CTs and non-contrast CTs. The second and third subsets include patients who had a chest CT scan obtained as part of the standard clinical care between March and May 2019, that is, the pre–COVID-19 pandemic phase. The radiologic reports of these studies were screened by a cardiothoracic radiologist with 12 years of clinical experience. The reports were labeled as having radiologic findings suggestive of infection or not. The adjudication was based on the presence of radiologic patterns usually associated with infection, including ground-glass opacities, consolidation, and nodular pattern, if such findings were described as fitting a differential diagnosis of infectious process based on the impression by the primary interpreting radiologists. These studies were consecutively selected to match the sex and age distribution of the COVID-19–positive subset and to represent two control groups with a balanced representation of chest CT showing findings suggestive of the infection (118) and findings not related to infection (118). The CC-CCII dataset was obtained from six different hospitals: 1) Sun Yat-sen Memorial Hospital and Third Affiliated Hospital of Sun Yat-sen University, 2) The first Affiliated Hospital of Anhui Medical University, 3) West China Hospital, 4) Nanjing Renmin Hospital, 5) Yichang Central People’s Hospital, and 6) Renmin Hospital of Wuhan University. The COVID-CTset dataset was from the Negin Medical Center, and the MosMedData dataset was from municipal hospitals in Moscow, Russia.

**TABLE 1:** Summary of data used in the study. These datasets include full volumetric CT scans of the patients.

Dataset	Origin	Description				Available at:
		Details	# Patients	# 3D scans	Label	
UTSW	UT Southwestern Medical Center	CT vendors: Phillips, Toshiba, GE Medical Systems	101	101	COVID-19 positive	*See footnote^1^
		Image resolution: 512 × 512	118	118	Infection (negative)	
		Pixel size range: 0.45 mm to 0.83 mm	118	118	Findings Unrelated to Infection (negative)	
		Slice thickness range: 0.9–3 mm				
		Format: DICOM						
China Consortium of Chest CT Image Investigation (CC-CCII) Dataset	Sun Yat-sen Memorial Hospital and Third Affiliated Hospital of Sun Yat-sen University, Guangzhou, China	CT vendor: unreported	929	1544	COVID-19 positive	http://ncov-ai.big.ac.cn/download
	The First Affiliated Hospital of Anhui Medical University, Anhui, China	Image resolution: mostly 512 × 512 (a few were 128 × 128)	964	1556	Common Pneumonia (negative)	
	West China Hospital, Sichuan, China	Pixel size range: unreported	849	1078	Normal Lung (negative)	
	Nanjing Renmin Hospital, Nanjing, China	Slice thickness range: 1–5 mm				
	Yichang Central People’s Hospital, Hubei, China							
	Renmin Hospital of Wuhan University, Wuhan, China							
COVID-CTset	Negin Medical Center, Sari, Iran	CT vendor: Siemens	95	281	COVID-19 positive	https://github.com/mr7495/COVID-CTset
		Image resolution: 512 × 512	282	1068	Normal lung (negative)	
		Pixel size range: unreported				
		Slice thickness range: unreported						
MosMedData	Municipal hospitals in Moscow, Russia	CT vendor: Toshiba	254	254	CT-0—not consistent with pneumonia (can include both COVID-19 positive and negative)	https://mosmed.ai/
		Image resolution: 512 × 512	684	684	CT-1—Mild (COVID-19 positive)	
		Pixel size range: unreported Slice thickness: 1 mm	125	125	CT-2—Moderate (COVID-19 positive)	
			45	45	CT-3—Severe (COVID-19 positive)	
			2	2	CT-4—Critical (COVID-19 positive)	

For consistency in training and testing the models in our study, we divided all the data into two classes: 1) COVID-19–positive and 2) COVID-19–negative patients. Note that MosMedData does not have conclusive negative–label patients, as CT-0 might contain both positive and negative patients. Accordingly, we omitted the CT-0 category from this study. Most scans in this study had a matrix size of 512 × 512 × n, where n is the variable number of slices. For the small number of scans that had a reduced matrix size, the images were linearly interpolated to match the 512 × 512 × n resolution.

**FIGURE 1:**
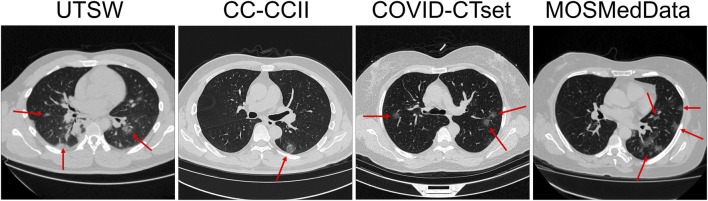
Slice view of example CTs from each dataset. Red arrows show patchy ground-glass opacities with round morphology, which are typical findings in COVID-19 pneumonia.

Most of the data were available in Hounsfield units (HU) or CT number (e.g. 0–4095). Some of the data in the CC-CCII dataset were provided in relative intensity values (e.g., 0–255). Because the data formatting varied across datasets, we performed clipping and normalization operations. First, if the data were displayed in HU, we clipped the minimum number to be −1,000 HU. For evaluation, the data were normalized from 0 to 1 prior to evaluation by the DL model. For training, multiple normalization methods were used as part of a data augmentation technique. The complete data augmentation is further described in the section *Training and Data Augmentation*. Figure 1 shows example CTs of COVID-19–positive patients from each dataset.

For training, validating, and testing the model, the positive labels of the UTSW dataset were randomly split into 73 train, 8 validation, and 20 test patients and scans (one 3D scan per patient). The positive labels of the CC-CCII dataset were randomly split into 669 train, 74 validation, and 186 test patients, or 1,110 train, 122 validation, 312 test scans. The positive labels of the COVID-CTset were randomly split into 68 train, 8 validation, and 19 test patients, or 201 train, 23 validation, and 57 test scans. The positive labels of MosMedData were randomly split into 616 train, 69 validation, and 171 test patients and scans (one 3D scan per patient; CT-0 category was omitted).

For the negative labels, the UTSW dataset was randomly split into 170 training, 18 validation, and 48 testing patients and scans (one 3D scan per patient). The CC-CCII dataset was randomly split into 1,305 train, 145 validation, and 363 test patients, or 1,891 train, 203 validation, and 540 test scans. The COVID-CTset was randomly split into 259 train, 29 validation, and 72 test patients, or 770 train, 84 validation, and 214 test scans.

### Model Architecture

**FIGURE 2:**
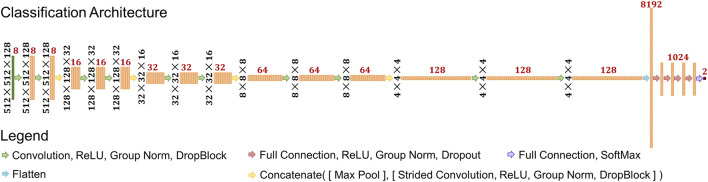
Schematic of deep learning architecture used in the study. Black numbers represent the feature shape of each layer prior to the flattening operation. Red numbers represent the number of features at each layer.

The model used in this study was a classification style convolutional neural network (CNN) model (LeCun et al., 1989; LeCun and Bengio, 1995; LeCun et al., 1998; LeCun et al., 1999), with specifics shown in Figure 2. The input shape was set to 512 × 512 × 128. There are five resolution levels of convolutions and four downsampling operations prior to the flattening operation. The downsampling size also varied each time and was set as (4,4,4), (4,4,2), (4,4,2), and (2,2,2), respectively. This converts the data shape from 512 × 512 × 128 to 4 × 4 × 4. At each resolution level, a series of operations consisting of convolution, Rectified Linear Unit activation (ReLU), Group Normalization (Wu and He, 2018), and DropBlock (Ghiasi et al., 2018) is applied twice, consecutively. The convolution kernel size varied at each resolution level: (3,3,3), (5,5,5), (5,5,3), (5,5,3), and (3,3,3). The number of filters, indicated by red numbers in Figure 2, at each convolution started at eight and doubled after each downsampling operation. After these operations, the feature data are flattened into a single vector of length 8,192. Then, a series of operations consisting of fully connected calculations, ReLU, Group Normalization, and Dropout (Srivastava et al., 2014) follows. This is performed a total of four times, calculating 1,024 features each time. Then, one more full connection is applied to reduce the data into two outputs, and a softmax operation is applied.

### Training and Data Augmentation

In total, nine models were trained in this study using the training and validation data outlined in *Data* and were split into two categories: 1) single dataset training and 2) multiple dataset training. We trained three models on a single dataset, one each on the UTSW, CC-CCII, and the COVID-CTset datasets. No model was trained on MosMedData by itself, since this dataset does not have any negative labels. For multiple dataset training, we trained six models with different combinations of datasets: 1) UTSW + CC-CCII, 2) UTSW + COVID-CTset, 3) CC-CCII + COVID-CTset, 4) UTSW + CC-CCII + COVID-CTset, 5) CC-CCII + COVID-CTset + MosMedData, and 6) UTSW + CC-CCII + COVID-CTset + MosMedData.

Some additional operations were applied to format and augment the CT data for model training. For CT data with less than 128 slices, slices of zeros were padded onto the CT slices until the total data volume had 128 slices. The number of slices superior and inferior to the CT data was uniformly and randomly decided at each iteration. For data with more than 128 slices, a random continuous volume of 128 slices was selected. The data were then normalized in one of two ways: 1) from 0 to 1, or 2) from 0 to $\begin{array}{l} \frac{max\;\left(data\right)}{2^{n}} \end{array}$, where *n* is the smallest integer possible while keeping 2^*n*^ larger than the maximum value in the CT volume. The normalization method was randomly chosen with a 50% chance during each training iteration. An additional step was applied to decide, at a 50% chance, whether this data would be fed into the model for training or if additional data augmentation would be applied. If yes to additional data augmentation, then the function randomly flipped, transposed, rotated, or scaled the data. For the flip augmentation, there was a 50% chance that it would individually apply a flip to each axis (row, column, and slice). For the transpose augmentation, there was a 50% chance that it would transpose the row and column of the data (no transpose operation was ever applied using the slice dimension). For the rotate augmentation, a random integer, {0,1,2,3}, was generated and multiplied against 90° to determine the rotation angle, then applied only on the row and column dimensions. For the scale augmentation, there was a 50% chance that a scaling factor was applied, and the scale was a uniform random number from 0 to 1.

Each model was trained for a total of 2,50,000 iterations—which is about 1,029, 83, 544, and 406 epochs for the UTSW, CC-CCII, COVID-CTSet, and MosMedData, respectively—using the Adam optimizer (Kingma and Ba, 2014) with a learning rate of 1 × 10^−5^. To prevent overfitting on the training data, the accuracy was evaluated on the validation data for every 500 iterations, and the instance of the model with the highest validation accuracy was saved as the final model for evaluation. The models were trained using NVIDIA V100 GPUs with 24 GB of memory.

### Evaluation

All nine of the trained models were evaluated on the test data of each dataset. For volumes with less than 128 slices, zero padding on the slices was evenly applied in the superior and inferior directions, to keep the data centered. For volumes greater than 128 slices, a sliding window technique was applied across the volume, and the model made multiple predictions. The number of slices in a patch was 128, and the stride size was 32 slices. The prediction with the highest COVID-19 probability was taken as the model’s final prediction.

A threshold was selected based on maximizing the prediction accuracy on the validation data and applied to the testing set. In the cases where the “optimal” threshold was a trivial value (e.g., threshold = 0 for MosMedData, which only has positive labels), we took the argmax of the output as the prediction instead. The true positives (TP), true negatives (TN), false positives (FP), and false negatives (FN) were counted, and a normalized confusion matrix was generated for each dataset. Averaged confusion matrices were calculated with and without MosMedData. An evenly weighted average was chosen.

Receiver operating characteristic (ROC) curves were calculated on the test data by varying the positive predictive threshold from 0 to 1, at 0.01 intervals. The area under the curve (AUC) was calculated to determine the overall performance of each model on each dataset. We additionally used the Bayesian approximate technique called Monte Carlo dropout (Gal and Ghahramani, 2016) to additionally estimate the uncertainty on the AUC. MosMedData was excluded from the ROC and AUC analyses, since it was missing negative labels.

## Results

Each model took about 5 days on average to train on a GPU. For nine models, this is equivalent to 45 GPU-days of training. Each model prediction takes an average of 0.53 s, which makes it very useful for near real-time applications.

**FIGURE 3:**
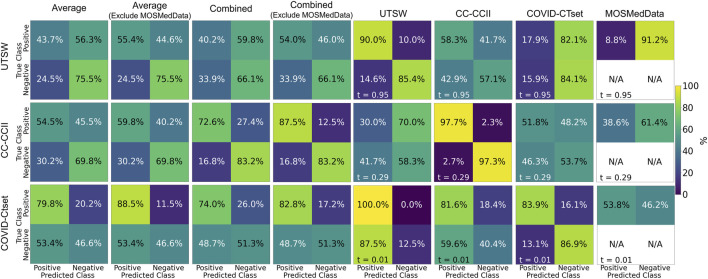
Confusion matrices on the test data for each of the models trained on a single dataset. Each row represents the datasets that the model was trained on, and each column represents the datasets that the model was evaluated on. Note that MosMedData does not have any negative label data. The labeling threshold used for each model is indicated on the lower left of each confusion matrix (*t* = #). The “Average” confusion matrix is an equally weighted average among each dataset, while the “Combined” confusion matrix is calculated from all samples from the datasets.

The single dataset models’ predictive accuracy $\left(\frac{TP+TN}{TP+TN+FP+FN}\right)$

on the test dataset is displayed in Figure 3. Overall, each model performed best on the dataset that it was trained on, with an accuracy as high as 0.97 for the CC-CCII model evaluated on the CC-CCII data. The model that performed the worst on its own dataset was COVID-CTset, with an accuracy of 0.86. The UTSW model had an accuracy of 0.87 on its own dataset. Since the test data were held out of the training and validation phase, it is a strong indicator that the model did not overfit to its specific training data. However, the models performed much more poorly when evaluated on a dataset they had not seen before, which signifies that the model did not generalize well to the new dataset type. The worst performance was the COVID-CTset model evaluated on the UTSW dataset, which had an accuracy of 0.38. All three models had poor performance on the MosMedData dataset.

**FIGURE 4:**
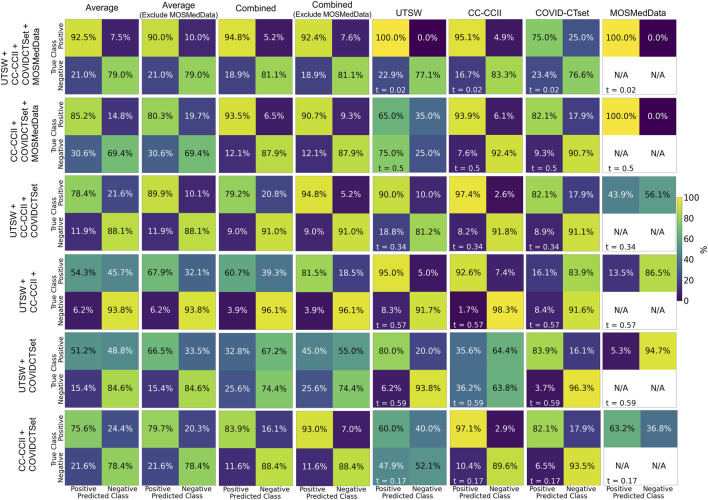
Confusion matrices on the test data for each of the models trained on multiple datasets. Each row represents the datasets that the model was trained on, and each column represents the datasets that the model was evaluated on. The labeling threshold used for each model is indicated on the lower left of each confusion matrix (*t* = #). The “Average” confusion matrix is an equally weighted average among each dataset, while the “Combined” confusion matrix is calculated from all samples from the datasets.

Figure 4 shows the confusion matrices of the performance of models trained on multiple datasets against the test data. The multiple dataset model that had the best accuracy when evaluated on the UTSW test set was the UTSW + CC-CCII model, with 0.93 accuracy. When evaluating the CC-CCII test set, the model with the best accuracy of 0.96 was the UTSW + CC-CCII model. When evaluating the COVID-CTset, the UTSW + COVID-CTset performed best, with an accuracy of 0.94. The best multiple dataset models outperformed their single dataset counterparts with regards to accuracy. However, these models still had poor accuracy when evaluated on a test dataset they have not seen before. For example, the model trained with the UTSW and COVID-CTset together had improved accuracies to 0.90 and 0.94 when evaluated on the test sets of the UTSW and COVID-CTset datasets, respectively. However, when evaluated on the CC-CCII dataset, the accuracy was 0.53. Including MosMedData in the model training improved the total average performance but did not improve the performance when evaluating models on the individual UTSW, CC-CCII, and COVID-CTset datasets.

**FIGURE 5:**
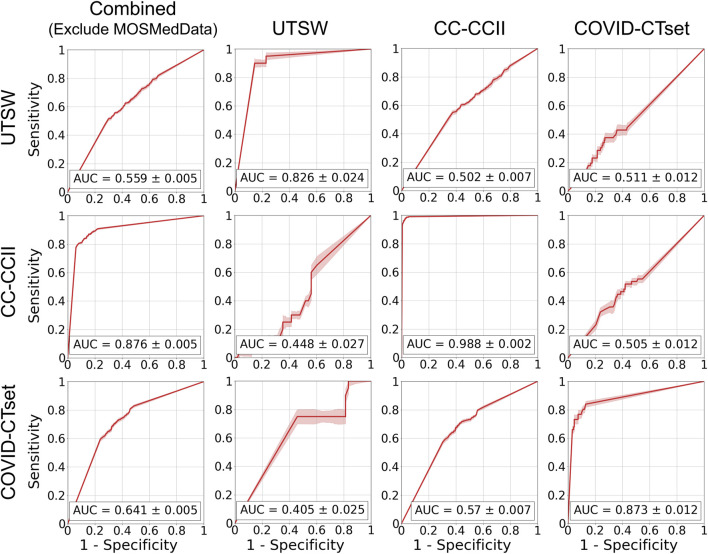
ROC curves on the test data for the models trained on single datasets. Each row represents the datasets that the model was trained on, and each column represents the datasets that the model was evaluated on. The error band and the error value in the reported AUC represent 1 standard deviation.

**FIGURE 6:**
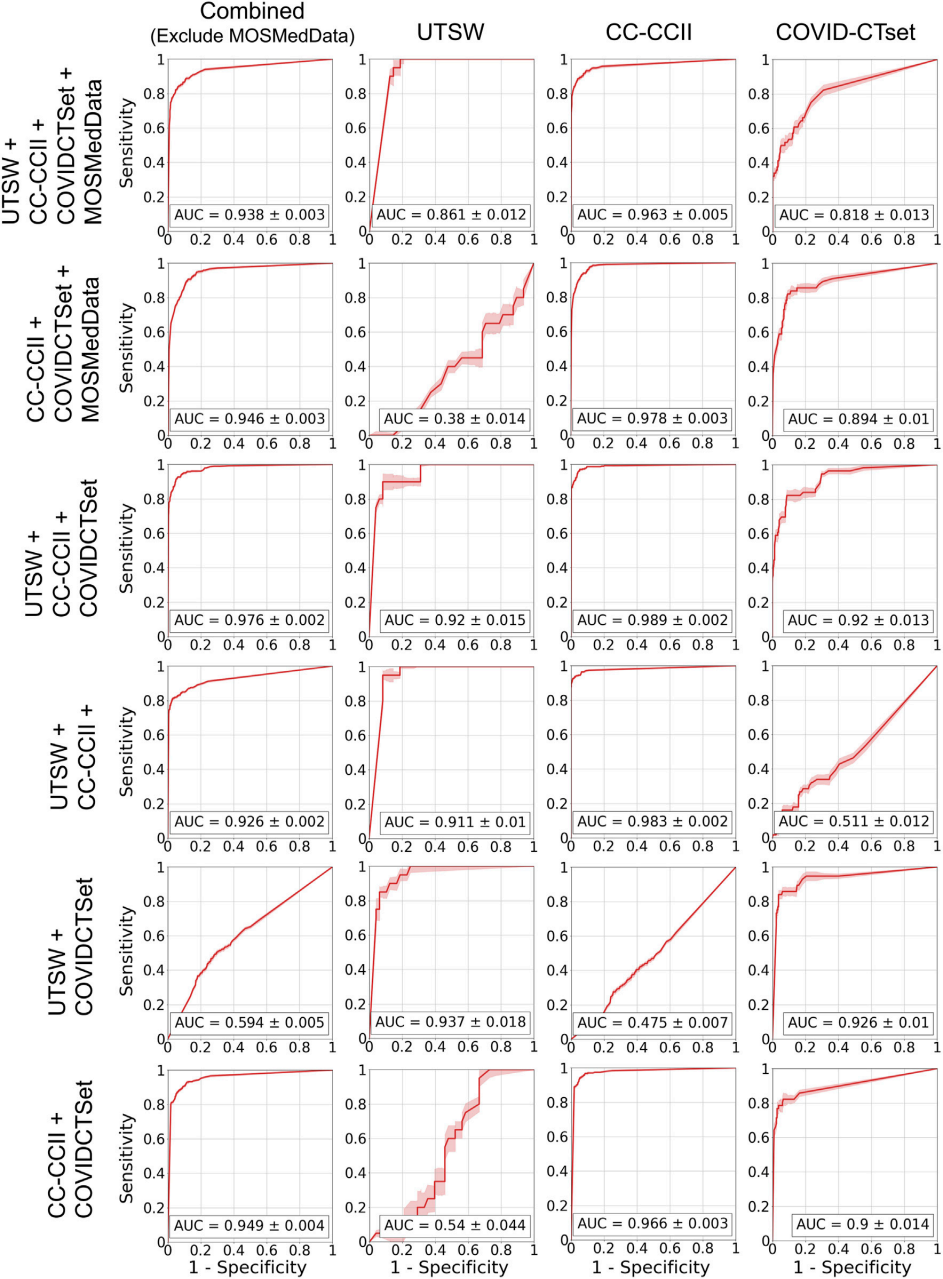
ROC curves on the test data for the models that trained on multiple datasets. Each row represents the datasets that the model was trained on, and each column represents the datasets that the model was evaluated on. The error band and the error value in the reported AUC represent 1 standard deviation.

Figure 5 shows the ROC curves of the single dataset models. The models, when evaluated on the same dataset that they were trained on, showed good AUCs (mean ± standard deviation) of 0.826 ± 0.024 (UTSW), 0.988 ± 0.002 (CC-CCII), and 0.873 ± 0.012 (COVID-CTset). The models performed considerably worse when evaluated on different datasets, with AUCs ranging from 0.405 to 0.570, which is close to just random guessing (i.e., AUC = 0.5). The ROC curves of the multiple dataset models are shown in Figure 6. For each dataset—UTSW, CC-CCII, and COVID-CTset—the best performing models were the UTSW + COVID-CTset (AUC = 0.937 ± 0.018), the UTSW + CC-CCII + COVID-CTset (AUC = 0.989 ± 0.002), and the UTSW + COVID-CTset (AUC = 0.926 ± 0.010) models, respectively. Since the test data were held entirely separate from the model development process, and used only for evaluation, this shows once again that the models did not overfit their own training data. Similar to the single dataset models, the multiple dataset models also performed poorly when predicting on datasets they had never seen before, with AUCs ranging from 0.380 to 0.540.

## Discussion

In this study, we demonstrate that our DL models can correctly identify patients that are COVID-19–positive with high accuracy, but only when the model was trained on the same datasets that it was tested on. Otherwise, the performance is poor—close to random guessing—which indicates that the model cannot easily generalize to an entirely new dataset distribution that it has never seen before for COVID-19 classification. Several data augmentation techniques were applied during training to prevent overfitting on the test set. In addition, the weights of the model that performed the best on the validation data with regards to accuracy were used as the final model. Dropout and DropBlock regularization were added to further prevent the model from overfitting.

We additionally observed that certain combined dataset models performed best for particular datasets in detecting patients who are positive for COVID-19. For example, we found that the highest performing model in the dataset from the UTSW dataset was obtained when the training step combined UTSW and CC-CCII datasets. This may have occurred due to the relatively low sample count in the UTSW dataset (73 positive, 170 negative patients for training); therefore, adding data samples from COVID-CTset improved with DL-model’s AUC from 0.826 to 0.937 on the UTSW dataset. Overall, the best-performing model for a particular dataset tended to be a multiple dataset model that included that same dataset in the training. When used properly, training on multiple datasets allows for having more training examples for the model to improve its overall feature extraction capabilities. There are many similarities between images, such as the texture and edges, which the model can learn from all the images. For example, it has been shown that models that pretrain on ImageNet (millions of images) can perform better on other classification tasks (Xie and Richmond, 2018). However, adding more data from different distributions into the training did not always monotonically improve the model's performance. For example, adding the CC-CCII data for training did not improve the model performance, with the AUC of 0.920 for the UTSW dataset. Adding MosMedData into the training lowered the performance of the model on the other three datasets. This is likely because the original intent of MosMedData was to train a model to categorize the severity of COVID-19 into five classes and, therefore, lacked negative labels. Without definitive negative labels, our models likely learned simply to identify the data source as MosMedData and compromised some of their learning capacity and performance to use the relevant imaging features for the predictions. This does serve as an important lesson in data collection: datasets from a particular healthcare center or region should be fully representative of the task at hand to be used in training. Simply collecting COVID-19–positive patients from one source and negative patients from a different source is likely to introduce an uncorrectable bias during training that led to a poor model performance.

We did include some state-of-the-art modules in our model, such as Group Normalization (Wu and He, 2018) and DropBlock (Ghiasi et al., 2018), that allowed for a high performance similar to other COVID-19 classification studies (Wang Z. et al., 2020; Ali et al., 2021; Song, 2021). Zech et al. investigated model generalizability in CT scans and found a similar conclusion, but a better one than a random guess on the unseen dataset (Zech et al., 2018). The major difference between this study and our study, where we only found a performance of around a random guess on an unseen test dataset, is that we investigated the generalizability of datasets across different countries around the world. The other study by Zech investigated datasets only from the United States, so it is likely that the differences in protocol, standards, and demographics between the datasets are much smaller than the dataset that we used. We intend to further investigate these differences and their impact across both intranation and internation datasets in a future study.

A potential source of bias may come from the discretization of data. While CT is typically stored in a 12-bit format, having 4,096 levels of discretization, some of the data in the CC-CCII dataset were stored in relative intensity values from 0 to 255. While we were careful with our normalization and data augmentation techniques, the more inherent coarseness in some of the data may have affected the model’s generalizability between datasets. When sharing or collecting datasets, it is of utmost importance to disclose the data’s exact format, as these can add more variability outside of the scanning protocol, quality, and demographics of a particular institution or region.

Between the UTSW dataset, CC-CCII dataset, and the COVID-CTset dataset, the CC-CCII dataset consistently yielded models that had the highest accuracy and AUC when evaluated on its own dataset. The exact reason for this is unknown, but it may be possible that there was an implicit bias within the dataset. For example, if one of the participating hospitals had a very different distribution of image quality, but also were a large provider of the data, then the model may have learned to simply distinguish that hospital specifically instead of the disease. However, the exact breakdown of where each individual scan originated from is not available. We will continue to investigate such cases and determine whether there was some sort of bias that allowed the CC-CCII dataset to yield models that gave high-accuracy values.

In contrast, the COVID-CTset dataset consistently yielded models that had the poorest performance. One potential reason is possibly its lack of variability of data to train on. For example, the UTSW dataset had COVID-19–negative scans that also included infected patients and the CC-CCII dataset had COVID-19–negative scans with common pneumonia. This may have helped the model further distinguish the nuances between COVID-19–positive and COVID-19–negative patients but with other presenting diseases. We plan to further identify and investigate these sources of biases in detail as part of a future study.

Although this study did not fully explore the possible techniques to improve robustness and prevent overfitting, it may serve as a baseline for future model generalization studies that use medical data for the clinical implementation of COVID-19–related classification models. We will continue to explore the limits of model generalization with respect to improving the algorithm and to the intra- and inter-source data variability, regarding the identification of COVID-19–positive patients by their medical data. As a whole, the deep learning models achieved a high performance on the unseen test set from the same distribution that they were trained on, which indicates that we did not have a typical overfitting problem with the training data. The low performance on datasets that the models had never seen before may actually be an indicator that the problem is not in the approach to the initial algorithm development—the problem may be the transfer and deployment of the algorithm to a new clinical setting. Creating a globally generalizable algorithm is a tall order, when people around the world have vastly different demographics and data collection protocols. With limited data and learning time, these AI algorithms are bound to fail when they encounter a unique data distribution they have never seen before. These results underscore the limited versatility of AI algorithms which may hamper the widespread adoption of AI algorithms for automated diagnosis of radiology images. This is in contrast to radiologists who in general can easily adapt to new clinical practices quickly. Perhaps we need to recalibrate our mindset with regard to the expectation for these AI algorithms—we should expect that these AI algorithms will always need to be fine-tuned to the local distribution when implemented and deployed in a specific clinical setting, then need to be retuned over time as distributions inevitably shift, either through demographic shifts or through the advancement of new treatment technologies. Transfer learning and continuous learning techniques (Torrey and Shavlik, 2010) are active fields of research and may become critical components to rapidly transfer, deploy, and maintain an AI model into the clinic.

AI tools designed for automatic identification of diseases on CT datasets, such as COVID-19, will only succeed if they can prove their robustness against a wide array of patient populations, scan protocols, and image quality. Notwithstanding, they hold the promise of becoming a powerful resource for identifying diseases, where time to detection is a critical variable. In the case of COVID-19, it is well known that many cases are asymptomatic, of which up to 54% will present abnormalities on chest CT (Inui et al., 2020). Thus, COVID-19 can be incidentally found on routine imaging. Timely identification of incidental cases of COVID-19 on chest CT by AI tools could lead to adequate prioritization of scans for reporting, resulting in prompt initiation of disease tracking and control measures. Moreover, the model architecture developed in this work can also serve as a template for similar tools tailored for detecting other clinical conditions.

The deep learning models were capable of identifying COVID-19–positive patients when the testing data was in the same dataset as the training data, whether the model was trained on a single dataset or on multiple datasets. However, we found a poor performance, close to random guessing, when models were evaluated on datasets that they had never seen. This is likely due to different factors, such as patient demographics, image acquisition methods/protocols, or diagnostic methods, causing a data shift between different countries’ data. This lack of generalization for the identification of COVID-19–positive patients may not necessarily mean that the models were trained poorly, but rather the distribution of the training data may be too different from the evaluation data. Transfer learning and continuous learning may become imperative tools for tuning and deploying a model in a new clinical setting.

## Data Availability

UTSW dataset is non-public. In accordance with HIPAA policy, access to the dataset will be granted on a case-by-case basis upon submission of a request to the corresponding authors and the institution. The DL models and related code developed in this study are available upon request for non-commercial research purposes.

## Footnotes

1 The UTSW dataset is nonpublic. In accordance with the HIPAA policy, access to the dataset will be granted on a case-by-case basis upon submission of a request to the corresponding authors and the institution.
